# Latent profile analysis and influencing factors of self-disclosure in patients after percutaneous coronary intervention for coronary heart disease: A cross-sectional study

**DOI:** 10.1097/MD.0000000000049276

**Published:** 2026-06-26

**Authors:** Yange Yang, Qiaoju Yang, Lijun Min, Jiayi Guan, Songbo Jia, Zhenzhen Wang

**Affiliations:** a School of Nursing (Nursing School of Smart Healthcare Industry), Henan University of Chinese Medicine, Zhengzhou, Henan, China; b Department of Cardiology, The First Affiliated Hospital of Henan University of Chinese Medicine, Zhengzhou, Henan, China.

**Keywords:** coronary heart disease, cross-sectional study, disease uncertainty, family resilience, latent profile analysis, percutaneous coronary intervention, self-disclosure

## Abstract

Self-disclosure influences psychological adjustment and disease management after percutaneous coronary intervention (PCI) for coronary heart disease (CHD), yet most evidence treats post-PCI patients as a homogeneous group. This study aimed to identify latent subgroups of self-disclosure and quantify the demographic, clinical, and psychosocial factors associated with subgroup membership. A cross-sectional survey was conducted among 270 post-PCI CHD patients in a tertiary hospital in Henan Province, China (April–October 2025). Patients completed the Distress Disclosure Index, Mishel Uncertainty in Illness Scale for Adults, and shortened Chinese Family Resilience Assessment Scale. Latent profile analysis was performed on the 12 Distress Disclosure Index items in Mplus 8.3. Variables significant in univariable analysis (*P* < .05) were entered into a multinomial multivariable logistic regression, with the lowest-disclosure profile as the reference. Three profiles were identified: low self-disclosure–difficulty without words (C1, n = 110, 40.74%), medium self-disclosure–emotional internalization (C2, n = 104, 38.52%), and high self-disclosure–lover talk (C3, n = 56, 20.74%); entropy was 0.952 and Nagelkerke pseudo-*R*^2^ was 0.621. Compared with C1, lower monthly household income was associated with reduced odds of C2 (income <3000 renminbi: odds ratio [OR] = 0.251, 95% confidence interval [CI]: 0.089–0.710, *P* = .009) and especially C3 (OR = 0.014, 95% CI: 0.002–0.088, *P* < .001). The absence of comorbid chronic conditions increased the odds of C2 (OR = 3.435, 95% CI: 1.211–9.749, *P* = .020) and C3 (OR = 7.652, 95% CI: 1.753–33.398, *P* = .007). Higher family resilience was positively associated with both C2 (OR = 1.032, 95% CI: 1.010–1.054, *P* = .004) and C3 (OR = 1.050, 95% CI: 1.015–1.087, *P* = .005), whereas higher disease uncertainty reduced the odds of C3 (OR = 0.895, 95% CI: 0.859–0.933, *P* < .001). Post-PCI CHD patients display 3 distinct self-disclosure profiles, with nearly 4 in 5 exhibiting moderate to low disclosure. Family resilience and disease uncertainty showed the largest associations with profile membership and may represent priority targets for tailored psychological care after PCI.

## 1. Introduction

The 2023 China Cardiovascular Health and Disease Report indicates that cardiovascular disease has become the leading cause of death among Chinese residents, surpassing malignant tumors and other diseases.^[1]^ The number of patients with coronary heart disease (CHD) is currently rising sharply, and the volume of percutaneous coronary intervention (PCI) procedures has grown accordingly.^[2]^ PCI relieves myocardial ischemia and hypoxia in the short term, but it does not fundamentally reverse atherosclerosis^[3]^; patients also require long-term medication, must avoid strenuous physical activity, and remain at risk of in-stent restenosis and no-reflow.^[4]^ These conditions impose persistent financial and physical burdens that frequently trigger anxiety, depression, and distress.

Self-disclosure refers to the process by which individuals reveal their thoughts and feelings to others.^[5]^ Sufficient self-disclosure facilitates emotional release, reduces negative affect, and supports active coping; in contrast, suppressed self-disclosure has been associated with reduced treatment adherence and poorer disease outcomes.^[6]^ Existing evidence suggests that self-disclosure is shaped by demographic characteristics,^[7]^ disease-related factors,^[8]^ and psychosocial factors.^[9]^ However, most prior studies adopt a variable-centered approach, using average scale scores to characterize patients as a homogeneous group, thereby obscuring meaningful interindividual heterogeneity. Latent profile analysis (LPA), a person-centered method, identifies unobserved subgroups based on response patterns and provides an intuitive view of how individuals cluster across multiple indicators. To our knowledge, no previous study has applied LPA to characterize self-disclosure in patients undergoing PCI for CHD.

The present study therefore had 2 specific objectives: first, to identify latent profiles of self-disclosure in patients after PCI for CHD; second, to examine which demographic, clinical, and psychosocial variables (specifically disease uncertainty and family resilience) are associated with profile membership and to quantify the magnitude of these associations. The findings may help inform the future development of profile-tailored psychological care after PCI.

## 2. Materials and methods

### 2.1. Study design and population

This study used a cross-sectional, convenience-sampling design and was reported in accordance with the Strengthening the Reporting of Observational Studies in Epidemiology statement for cross-sectional studies. Participants were patients who underwent PCI for CHD and were admitted to the Department of Cardiology of a tertiary hospital in Henan Province, China, between April and October 2025.

Inclusion criteria were as follows: meeting the diagnostic criteria for CHD established by the European Society of Cardiology^[10]^; age ≥18 years; a clinically stable condition after PCI^[11]^; and being conscious, with sufficient comprehension and communication ability to complete the questionnaire independently or with researcher assistance. Exclusion criteria were as follows: the presence of severe organic disease or major complications; a history of psychiatric disorder; or concurrent participation in another interventional study.

Sample size was determined using Kendall’s rule, which states that the number of participants should be 5 to 10 times the number of independent variables.^[12]^ Given 16 candidate variables and an anticipated 20% nonresponse rate, a minimum of 96 participants was required. To ensure stable LPA estimates and adequate statistical power for the regression analysis, 270 participants were ultimately enrolled.

The study was approved by the Institutional Review Board of the First Affiliated Hospital of Henan University of Chinese Medicine (Approval No. 2025HL-079-01), and written informed consent was obtained from every participant before data collection.

### 2.2. Measures

#### 2.2.1. General information questionnaire

The questionnaire collected age, sex, occupation, monthly household income, education level, marital status, number of comorbid chronic conditions, number of stents implanted, and number of prior interventional procedures. Because PCI is most often performed in the setting of acute coronary events, and patients may not reliably recall the precise onset of asymptomatic atherosclerosis, “duration of CHD” in this study was operationalized as the interval from the first physician-confirmed diagnosis of CHD (in the medical record) to the index hospital admission, recorded in months and verified against the electronic medical record rather than relying solely on patient recall.

#### 2.2.2. Mishel Uncertainty in Illness Scale for Adults (MUIS-A)

The MUIS-A was developed by Mishel^[13]^ and adapted for Chinese use by Xu and Huang.^[14]^ It contains 25 items across 2 dimensions, each scored on a 1 to 5 Likert scale (“strongly disagree” to “strongly agree”). Higher total scores indicate greater disease uncertainty. Cronbach α in the present sample was 0.891.

#### 2.2.3. Shortened Chinese version of the Family Resilience Assessment Scale (FRAS-C)

The original FRAS was developed by Sixbey^[15]^ and translated into a 32-item shortened Chinese version by Li et al^[16]^ in 2016. Items load on 3 dimensions and are scored on a 1 to 4 Likert scale; higher total scores indicate greater family resilience. Cronbach α in the present sample was 0.905.

#### 2.2.4. Distress Disclosure Index (DDI)

The DDI was developed by Kahn and Hessling^[17]^ in 2001 and revised for Chinese use by Li.^[18]^ It is unidimensional and comprises 12 items rated on a 5-point Likert scale; higher total scores indicate higher self-disclosure. Cronbach α in the present sample was 0.844.

### 2.3. Data collection procedure

Trained researchers screened consecutively admitted patients against the eligibility criteria, obtained written informed consent from those who met the criteria, and then administered the questionnaire battery in a private room. Before each survey, researchers delivered a standardized verbal introduction emphasizing that the questionnaire was anonymous, that there were no right or wrong answers, and that participation was voluntary. Researchers remained available throughout completion to clarify wording. Each questionnaire was checked for completeness immediately after collection; when items were missing, the patient was invited to complete them at that time. Questionnaires that remained incomplete because of patient fatigue, an interrupting clinical procedure, or refusal to continue, as well as questionnaires showing clearly patterned responses (for example, identical answers across items), were treated as invalid and excluded from analysis.

### 2.4. Statistical analysis

Analyses were conducted in IBM SPSS 26.0 (IBM Corp.) and Mplus 8.3 (Muthén & Muthén). All analyses were two-tailed and used a significance threshold of *P* < .05.

Common method bias was assessed before substantive analyses using Harman single-factor test^[19]^; an unrotated principal-component analysis was conducted on all self-report items, and common method bias was deemed acceptable when the largest factor explained <40% of the total variance.

LPA was performed in Mplus 8.3 using the 12 DDI items as manifest indicators. We restricted the manifest set to the DDI items because these items directly operationalize the latent construct of interest (self-disclosure); incorporating items from MUIS-A or FRAS-C would have conflated the latent construct with its hypothesized antecedents, thereby compromising the interpretability of the resulting profiles. We fitted models specifying 1 through 4 classes and compared them using the Akaike Information Criterion, Bayesian Information Criterion (BIC), and sample-size-adjusted BIC, with smaller values indicating better fit; entropy, with values closer to 1 indicating better classification quality; and the Lo-Mendell-Rubin adjusted likelihood ratio test and bootstrapped likelihood ratio test, where a significant test (*P* < .05) indicated that the k-class model fitted the data better than the (*k* − 1)-class model. The final model was selected jointly on the basis of statistical fit indices, entropy, average posterior membership probability, sample size in the smallest class, and substantive interpretability.

Continuous variables that violated the normality assumption (Shapiro–Wilk *P* < .05) were summarized as median (Q1, Q3); categorical variables were summarized as frequencies and percentages. Univariable comparisons of demographic, clinical, and psychosocial variables across the 3 latent classes were conducted with the chi-square test for categorical variables and the Kruskal–Wallis *H* test for continuous non-normal variables.

Variables that reached *P* < .05 in the univariable analyses were entered simultaneously (enter method) as candidate covariates into a multinomial multivariable logistic regression model, with profile membership as the dependent variable and the lowest-disclosure profile (C1) as the reference category. Categorical covariates were dummy-coded, with the reference category specified for each variable. Before modeling, multicollinearity was inspected using the variance inflation factor (VIF), with VIF < 5 considered acceptable. Adjusted associations are reported as odds ratios (ORs) with 95% confidence intervals (CIs). Model performance was summarized using the Nagelkerke pseudo-*R*^2^ as an indication of how much variation in profile membership the predictors explained.

## 3. Results

### 3.1. Common method bias

Harman single-factor test extracted 22 components with eigenvalues >1; the first unrotated component explained 19.51% of the variance, well below the conventional 40% threshold. Common method bias was therefore unlikely to substantially confound the findings.^[19]^

### 3.2. Sample characteristics

A total of 295 questionnaires were distributed, and 270 valid questionnaires were retained (effective response rate, 91.53%). The sample comprised 164 men (60.74%) and 106 women (39.26%); 74 participants (27.40%) had completed primary school or below, 72 (26.67%) junior high school, 71 (26.30%) senior high school or vocational school, and 53 (19.63%) college or above. Detailed characteristics are presented in Table 2.

### 3.3. Scale scores

Patients had a median DDI total score of 36.00 (Q1 = 26.00; Q3 = 45.00), corresponding to a moderate level of self-disclosure. The median MUIS-A total was 69.00 (Q1 = 60.00; Q3 = 83.25), and the median FRAS-C total was 84.00 (Q1 = 63.00; Q3 = 96.00).

### 3.4. Latent profile selection

Models with 1 through 4 classes were fitted to the 12 DDI items. The Akaike Information Criterion, BIC, and sample-size-adjusted BIC decreased monotonically as the number of classes increased; entropy exceeded 0.80 from the 2-class model onward. The Lo-Mendell-Rubin adjusted likelihood ratio test *P*-value remained significant for the 2- and 3-class solutions but became nonsignificant when moving from 3 to 4 classes (*P* = .0743), indicating no additional support for a 4-class structure. Considering statistical fit indices together with the substantive interpretability of each class and the requirement that no class be too small for inference, the 3-class model was selected. In this final model, the average posterior membership probability ranged from 96.9% to 98.4%, indicating reliable classification (Table 1).

**Table 1:** Model fit indices for latent profile analysis of self-disclosure (n = 270).

Model	AIC	BIC	aBIC	Entropy	LMRT	BLRT	Category probability (%)
1	10,930.911	11,017.274	10,941.177				
2	9998.761	10,131.903	10,014.588	0.905	<0.001	<0.001	0.482/0.519
3	9661.723	9841.645	9683.111	0.952	<0.001	<0.001	0.412/0.381/0.207
4	9542.313	9769.014	9569.261	0.960	0.0743	<0.001	0.407/0.063/0.144/0.385

Model labels 1 to 4 refer to the number of latent classes. Lower AIC/BIC/aBIC and higher entropy indicate better statistical fit; significant LMRT/BLRT (*P* < .05) indicates that the k-class model fits significantly better than the (*k* − 1)-class model.

aBIC *=* sample-size-adjusted BIC, AIC *=* Akaike Information Criterion, BIC *=* Bayesian Information Criterion, BLRT *=* bootstrapped likelihood ratio test, LMRT *=* Lo-Mendell-Rubin adjusted likelihood ratio test.

### 3.5. Profile description and naming

**Figure 1.**
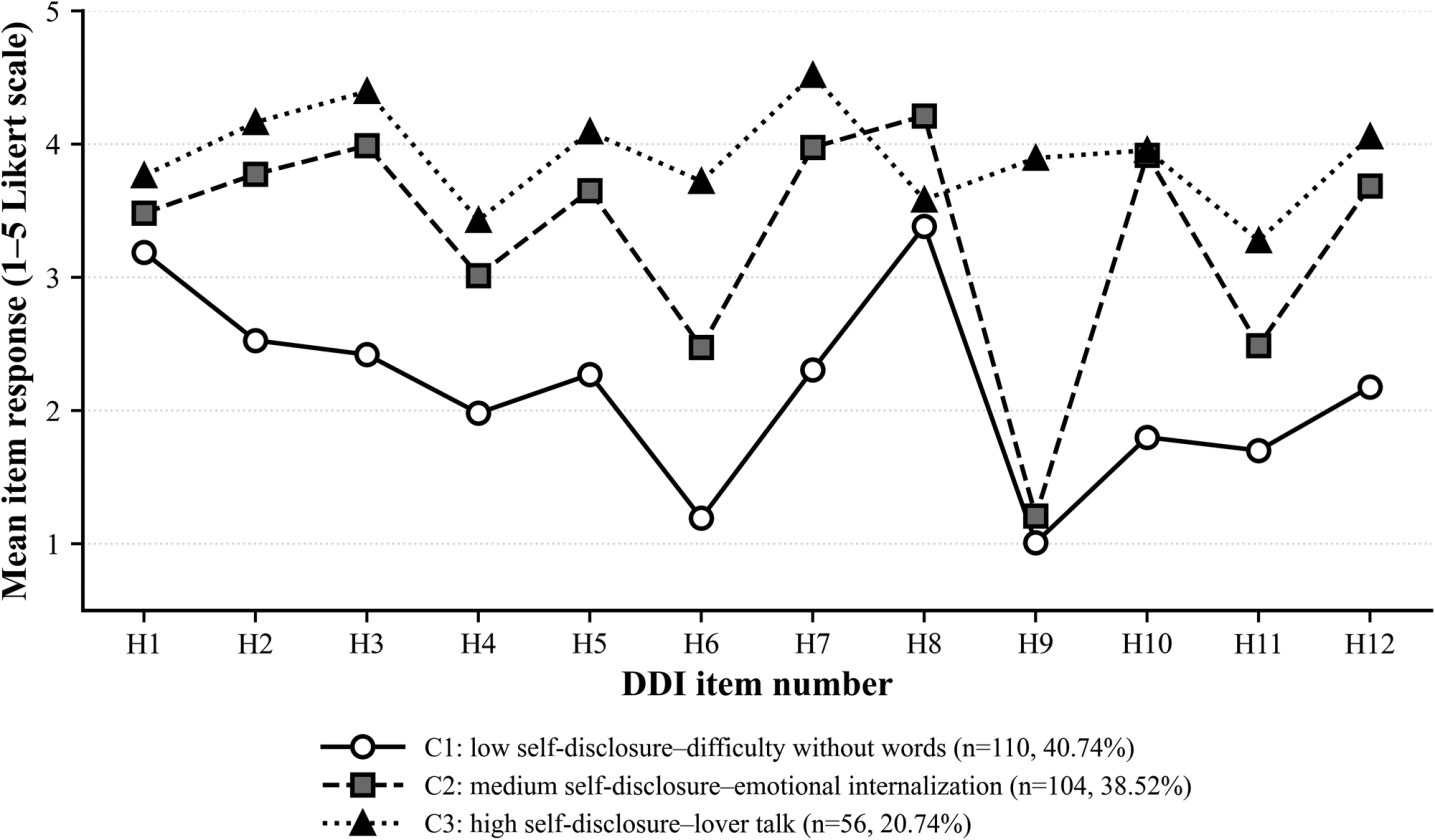
Latent profile patterns of self-disclosure among 270 post-PCI CHD patients. The horizontal axis lists the 12 items of the DDI in their original order; the vertical axis represents the within-class mean item response on the original 1 to 5 Likert scale. C1 = low self-disclosure–difficulty without words group (n = 110, 40.74%); C2 = medium self-disclosure–emotional internalization group (n = 104, 38.52%); C3 = high self-disclosure–lover talk group (n = 56, 20.74%). CHD = coronary heart disease, DDI = Distress Disclosure Index, PCI = percutaneous coronary intervention.

The 3-class profile is shown in Figure 1, where the y-axis (“Mean item response”) represents the within-class mean of each DDI item on the original 1 to 5 Likert scale, and the x-axis lists the 12 DDI items in their original order.

C1 (n = 110, 40.74%) had item means between 1.009 and 3.383, with the lowest values on Item 6 (“I seek others to discuss my problems”) and reverse-scored Item 9 (“When facing difficulties, I rarely discuss them with others”); patients in this class were rarely willing to seek discussion when facing difficulties, and the class was therefore named the **low self-disclosure–difficulty without words group**. C2 (n = 104, 38.52%) had item means between 1.209 and 4.213, with moderate scores on most items but a higher score on reverse-scored Item 8 (“When I feel sad, I am least willing to confide in others”), indicating a tendency to internalize negative affect rather than express it; this class was named the **medium self-disclosure–emotional internalization group**. C3 (n = 56, 20.74%) had item means between 3.277 and 4.396 (the highest of the 3 classes); Item 7 (“When I feel down, I talk to my lover”) received the highest score, suggesting that emotional support was sought predominantly within the spousal relationship. This class was named the **high self-disclosure–lover talk group**.

### 3.6. Univariable analysis

Differences across the 3 classes were statistically significant (*P* < .05) for age, education level, monthly household income, number of comorbid chronic conditions, number of interventional procedures, MUIS-A score, and FRAS-C score. Sex, occupation, marital status, and number of stents implanted did not differ across classes (*P* > .05). Detailed values are presented in Table 2.

**Table 2:** Univariable comparison of demographic, clinical, and psychosocial variables across the 3 latent self-disclosure profiles (n = 270).

Variables	C1	C2	C3	Total	χ^2^/*F*	*P*
Sex					2.544	.280
Male	73 (66.36)	60 (57.69)	31 (55.36)	164		
Female	37 (33.64)	44 (42.31)	25 (44.64)	106		
Age (yr)					14.805	.022
18–44	7 (6.36)	6 (5.77)	2 (3.57)	15		
45–59	31 (28.18)	42 (40.38)	17 (30.36)	90		
60–74	62 (56.36)	47 (45.19)	23 (41.07)	132		
≥75	10 (9.09)	9 (8.65)	14 (25.00)	33		
Education level					43.091	<.001
Elementary school or below	47 (42.73)	20 (19.23)	7 (12.50)	74		
Junior high school	25 (22.73)	38 (36.54)	9 (16.07)	72		
High school or vocational school	28 (25.45)	26 (25.00)	17 (30.36)	71		
College or above	10 (9.09)	20 (19.23)	23 (41.07)	53		
Marital status					0.241	1.000
Single	3 (2.73)	3 (2.88)	1 (1.79)	7		
Married	107 (97.27)	101 (97.12)	55 (98.21)	263		
Primary caregiver					1.015	.907
Spouse	62 (56.36)	61 (58.65)	31 (55.36)	154		
Parents or children	41 (37.27)	39 (37.50)	21 (37.50)	101		
Other	7 (6.36)	4 (3.85)	4 (7.14)	15		
Residence					0.064	.968
Rural	49 (44.55)	48 (46.15)	25 (44.64)	122		
Urban	61 (55.45)	56 (53.85)	31 (55.36)	148		
Occupation					9.165	.164
Employed	25 (22.73)	15 (14.42)	8 (14.29)	48		
Retired	25 (22.73)	32 (30.77)	21 (37.50)	78		
Self-employed or farmer	48 (43.64)	43 (41.35)	25 (44.64)	116		
Unemployed	12 (10.91)	14 (13.46)	2 (3.57)	28		
Monthly household income (RMB)					31.049	<.001
<3000	52 (47.27)	38 (36.54)	4 (7.14)	94		
3000–4999	42 (38.18)	36 (34.62)	29 (51.79)	107		
≥5000	16 (14.55)	30 (28.85)	23 (41.07)	69		
Health insurance type					2.129	.370
Self-pay	6 (5.45)	2 (1.92)	1 (1.79)	9		
Medical insurance	104 (94.55)	102 (98.08)	55 (98.21)	261		
Body mass index					7.572	.239
<18.5	0 (0.00)	3 (2.88)	1 (1.79)	4		
18.5–23.9	38 (34.55)	41 (39.42)	27 (48.21)	106		
24–27.9	46 (41.82)	38 (36.54)	21 (37.50)	105		
≥28	26 (23.64)	22 (21.15)	7 (12.50)	55		
Number of comorbid chronic conditions					25.250	<.001
0	10 (9.09)	30 (28.85)	21 (37.50)	61		
1	53 (48.18)	43 (41.35)	26 (46.43)	122		
≥2	47 (42.73)	31 (29.81)	9 (16.07)	87		
Duration of coronary heart disease					7.150	.128
<1 mo	29 (26.36)	28 (26.92)	22 (39.29)	79		
1–11 mo	21 (19.09)	25 (24.04)	5 (8.93)	51		
≥12 mo	60 (54.55)	51 (49.04)	29 (51.79)	140		
Number of stents implanted					9.161	.057
≤1	43 (39.09)	62 (59.62)	29 (51.79)	134		
2	28 (25.45)	17 (16.35)	11 (19.64)	56		
≥3	39 (35.45)	25 (24.04)	16 (28.57)	80		
Number of interventional procedures					39.729	<.001
1	71 (64.55)	33 (31.73)	22 (39.29)	126		
2	34 (30.91)	62 (59.62)	20 (35.71)	116		
≥3	5 (4.55)	9 (8.65)	14 (25.00)	28		
Disease uncertainty (score)	82.00 (67.75, 98.25)	69.50 (63.00, 75.00)	57.00 (49.00, 64.00)		47.629	<.001
Family resilience (score)	70.50 (59.00, 89.00)	88.00 (61.25, 97.75)	96.00 (84.00, 101.00)		70.910	<.001

Continuous variables are reported as median (Q1, Q3); categorical variables are reported as frequency (column %). Group comparisons used the Kruskal–Wallis *H* test for continuous variables and the chi-square (χ^2^) test for categorical variables. C1 = low self-disclosure–difficulty without words group; C2 = medium self-disclosure–emotional internalization group; C3 = high self-disclosure–lover talk group.

RMB = renminbi.

### 3.7. Multivariable logistic regression

All 7 variables that reached significance in the univariable analyses were entered into the multinomial multivariable logistic regression model; VIF values for all candidate predictors were below 5, indicating no severe multicollinearity. Using C1 as the reference, the final model achieved a Nagelkerke pseudo-*R*^2^ of 0.621, indicating that the predictors explained approximately 62% of the variation in profile membership. Adjusted ORs with 95% CIs are reported in Table 3.

**Table 3:** Multinomial multivariable logistic regression for self-disclosure profile membership (n = 270).

Variables	Reference	C2:C1						C3:C1					
		*B*	SE	Wald χ^2^	*P*	OR	95% CI	*B*	SE	Wald χ^2^	*P*	OR	95% CI
Age (yr)													
18–44	≥75	−0.209	0.934	0.050	.823	0.811	0.130–5.064	−2.490	1.393	3.194	.074	0.083	0.005–1.272
45–59		0.173	0.636	0.074	.786	1.189	0.341–4.138	−1.746	0.905	3.723	.054	0.174	0.030–1.028
60–74		−0.235	0.590	0.159	.691	0.791	0.249–2.513	−1.378	0.831	2.750	.097	0.252	0.049–1.285
Education level													
Elementary school or below	College or above	−1.599	0.626	6.521	.011	0.202	0.059–0.690	−2.156	0.837	6.632	.010	0.116	0.022–0.597
Junior high school		−0.280	0.613	0.208	.649	0.756	0.227–2.516	−1.021	0.814	1.572	.210	0.360	0.073–1.777
High school or vocational school		−0.529	0.619	0.732	.392	0.589	0.175–1.981	−0.728	0.799	0.831	.362	0.483	0.101–2.310
Monthly household income (RMB)													
<3000	≥5000	−1.382	0.530	6.793	.009	0.251	0.089–0.710	−4.240	0.921	21.170	<.001	0.014	0.002–0.088
3000–4999		−1.266	0.499	6.425	.011	0.282	0.106–0.750	−1.531	0.641	5.703	.017	0.216	0.062–0.760
Number of comorbid chronic conditions													
0	≥2	1.234	0.532	5.379	.020	3.435	1.211–9.749	2.035	0.752	7.327	.007	7.652	1.753–33.398
1		−0.122	0.382	0.102	.750	0.885	0.419–1.872	−0.008	0.621	0.000	.990	0.993	0.294–3.352
Number of interventional procedures													
1	≥3	−1.742	0.713	5.970	.015	0.175	0.043–0.709	−2.631	0.855	9.461	.002	0.072	0.013–0.385
2		−0.102	0.705	0.021	.885	0.903	0.227–3.596	−1.520	0.852	3.182	.074	0.219	0.041–1.162
Family resilience		0.031	0.011	8.119	.004	1.032	1.010–1.054	0.049	0.017	7.991	.005	1.050	1.015–1.087
Disease uncertainty		−0.039	0.011	12.226	<.001	0.961	0.940–0.983	−0.110	0.021	27.878	<.001	0.895	0.859–0.933

C1 = low self-disclosure–difficulty without words group; C2 = medium self-disclosure–emotional internalization group; C3 = high self-disclosure–lover talk group; Reference category = C1 (low self-disclosure–difficulty without words group). Model adjusted for all variables that reached *P* < .05 in the univariable analyses; VIF for every predictor was below 5. Nagelkerke pseudo-*R*^2^ = 0.621.

*B* = regression coefficient, CI = confidence interval, OR = odds ratio, RMB = renminbi, SE = standard error, VIF = variance inflation factor.

For C2 versus C1, the odds of membership in the medium-disclosure profile were lower among patients with elementary school or below versus college or above (OR = 0.202, 95% CI: 0.059–0.690, *P* = .011), monthly household income <3000 renminbi (RMB) versus ≥5000 RMB (OR = 0.251, 95% CI: 0.089–0.710, *P* = .009), monthly household income 3000 to 4999 RMB versus ≥5000 RMB (OR = 0.282, 95% CI: 0.106–0.750, *P* = .011), and 1 prior interventional procedure versus ≥3 procedures (OR = 0.175, 95% CI: 0.043–0.709, *P* = .015). In contrast, having no comorbid chronic conditions versus ≥2 (OR = 3.435, 95% CI: 1.211–9.749, *P* = .020) and higher family resilience (OR = 1.032, 95% CI: 1.010–1.054, *P* = .004) increased the odds of C2 membership, whereas higher disease uncertainty decreased those odds (OR = 0.961, 95% CI: 0.940–0.983, *P* < .001). For C3 versus C1, the odds of membership in the high-disclosure profile were lower among patients with elementary school or below versus college or above (OR = 0.116, 95% CI: 0.022–0.597, *P* = .010), monthly household income <3000 RMB versus ≥5000 RMB (OR = 0.014, 95% CI: 0.002–0.088, *P* < .001), monthly household income 3000 to 4999 RMB versus ≥5000 RMB (OR = 0.216, 95% CI: 0.062–0.760, *P* = .017), and 1 prior interventional procedure versus ≥3 procedures (OR = 0.072, 95% CI: 0.013–0.385, *P* = .002). Having no comorbid chronic conditions versus ≥2 (OR = 7.652, 95% CI: 1.753–33.398, *P* = .007) and higher family resilience (OR = 1.050, 95% CI: 1.015–1.087, *P* = .005) increased the odds of C3 membership, whereas higher disease uncertainty decreased those odds (OR = 0.895, 95% CI: 0.859–0.933, *P* < .001).

## 4. Discussion

### 4.1. Heterogeneity in self-disclosure

This study identified 3 latent profiles of self-disclosure in post-PCI CHD patients. Almost 4 in 5 patients (79.26%) belonged to a moderate- or low-disclosure profile, a pattern broadly consistent with Tao et al’s findings in heart failure patients.^[20]^

The largest profile (C1, 40.74%) showed the lowest willingness to discuss disease-related concerns. These patients may view disease coping as a personal responsibility and may prefer not to share treatment-related feelings with others. Clinical priority should therefore be given to identifying low-disclosure patients early. Practical strategies include structured psychoeducation about the value of disclosure, encouragement to use verbal communication, written expression, or supportive group therapy, and the establishment of peer support groups in which patients with comparable experiences can share emotions and information.

The second profile (C2, 38.52%) showed a moderate level of disclosure together with reluctance to share negative emotions. This pattern is consistent with the cultural notion of “sharing only good news,” in which expressing vulnerability is perceived as a burden on the family. Repeated family education workshops can help relatives understand patient needs and remove the assumption that emotional sharing is burdensome, thereby allowing patients to release internalized stress.

The third profile (C3, 20.74%) showed high overall disclosure that was concentrated toward the spouse, indicating that the spousal bond plays a central role in psychological adjustment. Strong intimate ties provide timely emotional response and understanding, both of which support psychological resilience.^[21]^ Healthcare providers should encourage spouses and other close family members to maintain a nonjudgmental environment in which the depth and frequency of disclosure can grow.

### 4.2. Influencing factors

#### 4.2.1. Monthly household income

Compared with the ≥5000 RMB group, patients with monthly household income <3000 RMB and 3000 to 4999 RMB had substantially lower odds of belonging to C2 (OR = 0.251 and 0.282, respectively), and income <3000 RMB also markedly reduced the odds of belonging to C3 (OR = 0.014). This finding is consistent with Du et al.^[22]^ Because PCI is associated with substantial out-of-pocket costs (about 30,000–50,000 RMB) and long-term pharmacotherapy,^[23,24]^ lower-income patients may be more inclined to internalize emotion rather than share it, partly out of concern that disclosure adds to family financial strain. In practice, clinicians should systematically assess economic capacity, openly discuss treatment costs, propose more cost-effective regimens when clinically equivalent, and provide information on social insurance and chronic disease coverage.

#### 4.2.2. Education

Lower educational attainment was associated with the low-disclosure profile, in line with Tang et al.^[25]^ Patients with limited literacy may have less access to disease information and may struggle to articulate concerns. Diversified education materials (handbooks, departmental lectures, short videos, social media-based health communication) and clinician empathy are valuable in supporting this subgroup.^[26]^

#### 4.2.3. Number of comorbid conditions

Compared with patients who had ≥2 comorbid chronic conditions, those with no comorbid chronic condition had 3.435-fold higher odds of belonging to C2 and 7.652-fold higher odds of belonging to C3. Patients with multiple comorbidities face heavier polypharmacy and a higher financial burden, more bodily symptoms, and stronger feelings of helplessness; together, these factors reinforce avoidance and silence. Clinical management should integrate optimization of combined medication regimens, structured post-discharge symptom monitoring, and digital tools for cardiovascular secondary prevention.^[27]^ Online disclosure groups can also offer a partially anonymized space in which patients are more comfortable sharing sensitive concerns.^[28–30]^

#### 4.2.4. Number of interventional procedures

Patients undergoing only 1 procedure had markedly lower odds of belonging to C2 (OR = 0.175) or C3 (OR = 0.072) than patients who had undergone ≥3 procedures, consistent with Zhou et al^[31]^; novelty, perceived risk, and uncertainty around prognosis appear to amplify suppression. Repeated intervention may also signify recurrence and lead to silence, suggesting a potentially nonlinear relationship between procedure count and disclosure. Differentiated education matched to procedural experience, mindfulness-based interventions,^[32]^ and cognitive-behavioral approaches^[33]^ may help reduce psychological stress and encourage open dialogue.

#### 4.2.5. Disease uncertainty

A Higher MUIS-A score was associated with lower odds of belonging to either higher-disclosure profile, with the stronger inverse association observed for C3 versus C1 (C2 vs C1: OR = 0.961 per 1-point increase; C3 vs C1: OR = 0.895 per 1-point increase). Greater uncertainty reduces patients’ sense of cognitive clarity and control, leading to avoidance.^[34]^ This is consistent with Zhang et al,^[35]^ who reported that limited disease knowledge, complex treatment, and concern about restenosis amplify uncertainty. Clinicians should reduce uncertainty through structured information support that explains pathogenesis, the principles of PCI, and prognosis,^[36]^ and should explicitly link healthy lifestyle changes to disease outcomes.

#### 4.2.6. Family resilience

Higher family resilience was consistently associated with both higher-disclosure profiles: each 1-point increase in FRAS-C score raised the odds of belonging to C2 by 3.2% (OR = 1.032) and to C3 by 5.0% (OR = 1.050). These findings echo prior work in elderly people living with HIV^[37]^ and indicate that the family functions as a primary support system after PCI.^[38]^ Clinicians should evaluate family resilience routinely and integrate spouses and other close relatives into the psychological care plan, including medication adherence, exercise, follow-up visits, and lifestyle modification.

### 4.3. Magnitude and practical implications

When the magnitude of associations is considered together with statistical significance, categorical and continuous predictors should not be ranked using raw ORs alone because their unit scales differ. We therefore interpreted categorical contrasts and 1-point continuous effects separately. Among categorical variables, low monthly household income (<3000 RMB vs ≥5000 RMB: OR = 0.251 for C2 vs C1 and OR = 0.014 for C3 vs C1), absence of comorbid chronic conditions (0 vs ≥2: OR = 3.435 for C2 vs C1 and OR = 7.652 for C3 vs C1), and 1 interventional procedure versus ≥3 procedures (OR = 0.175 for C2 vs C1 and OR = 0.072 for C3 vs C1) showed the largest contrasts. Among continuous psychosocial variables, higher family resilience was consistently associated with both higher-disclosure profiles, whereas higher disease uncertainty was most strongly associated with reduced odds of C3 membership. The Nagelkerke pseudo-*R*^2^ of 0.621 indicates that the model explains a substantial, although not exhaustive, proportion of variation, leaving room for additional psychosocial mediators (for example, perceived social support and dyadic coping) in future work. Clinically, the present results support a tiered psychological care strategy: routine identification of low-disclosure patients on admission; family resilience-oriented intervention as a major psychosocial lever; and targeted information support for patients with high disease uncertainty.

## 5. Limitations

The present study has several limitations. First, the sample was drawn by convenience from a single tertiary hospital in Zhengzhou, Henan, which limits generalizability to patients in other regions or hospitals at different levels of care. Second, the cross-sectional design captures associations at a single time point and cannot establish causal direction. Third, all measurements relied on self-report, which is subject to information and social desirability bias; future studies should consider triangulating self-report with informant ratings or behavioral indices. Fourth, although Nagelkerke pseudo-*R*^2^ indicated that the predictors explained roughly 62% of profile membership variance, residual unexplained variance suggests that mediators not modeled here, such as perceived social support and dyadic coping, deserve attention. Future research should pursue multicenter recruitment, longitudinal follow-up, and intervention designs.

## 6. Conclusion

This cross-sectional study demonstrates clear heterogeneity of self-disclosure among post-PCI CHD patients, with 3 latent profiles ranging from low disclosure with verbal difficulty to high disclosure focused on the spouse. Monthly household income, education, number of comorbid conditions, number of interventional procedures, family resilience, and disease uncertainty were all independently associated with profile membership; family resilience and disease uncertainty showed the largest effect sizes. These findings argue for routine assessment of self-disclosure after PCI and for tailored, profile-specific psychological support that prioritizes family resilience-oriented intervention.

## Acknowledgments

The authors thank all patients who took part in this study and the medical and nursing staff of the Department of Cardiology at the First Affiliated Hospital of Henan University of Chinese Medicine for their support during data collection.

## Author contributions

**Conceptualization:** Qiaoju Yang, Yange Yang.

**Data curation:** Yange Yang, Jiayi Guan, Lijun Min.

**Formal analysis:** Yange Yang, Songbo Jia, Zhenzhen Wang.

**Investigation:** Yange Yang, Lijun Min, Jiayi Guan.

**Methodology:** Qiaoju Yang, Yange Yang.

**Project administration:** Qiaoju Yang.

**Resources:** Qiaoju Yang, Songbo Jia, Zhenzhen Wang.

**Software:** Yange Yang.

**Supervision:** Qiaoju Yang.

**Validation:** Lijun Min, Songbo Jia, Zhenzhen Wang.

**Visualization:** Yange Yang.

**Writing – original draft:** Yange Yang.

**Writing – review & editing:** Qiaoju Yang, Lijun Min, Jiayi Guan, Songbo Jia, Zhenzhen Wang.
